# Schools as Moderators of Genetic Associations with Life Course Attainments: Evidence from the WLS and Add Health

**DOI:** 10.15195/v5.a22

**Published:** 2018-08-02

**Authors:** Sam Trejo, Daniel W Belsky, Jason D. Boardman, Jeremy Freese, Kathleen Mullan Harris, Pam Herd, Kamil Sicinski, Benjamin W. Domingue

**Affiliations:** a) Stanford University; b) Duke University; c) University of Colorado Boulder; d) University of North Carolina at Chapel Hill; e) University of Wisconsin-Madison

**Keywords:** polygenic score, educational attainment, GxE, schools

## Abstract

Genetic variants identified in genome-wide association studies of educational attainment have been linked with a range of positive life course development outcomes. However, it remains unclear whether school environments may moderate these genetic associations. We analyze data from two biosocial surveys that contain both genetic data and follow students from secondary school through mid- to late life. We test if the magnitudes of the associations with educational and occupational attainments varied across the secondary schools that participants attended or with characteristics of those schools. Although we find little evidence that genetic associations with educational and occupational attainment varied across schools or with school characteristics, genetic associations with any postsecondary education and college completion were moderated by school-level socioeconomic status. Along similar lines, we observe substantial between-school variation in the average level of educational attainment students achieved for a fixed genotype. These findings emphasize the importance of social context in the interpretation of genetic associations. Specifically, our results suggest that though existing measures of individual genetic endowment have a linear relationship with years of schooling that is relatively consistent across school environments, school context is crucial in connecting an individual’s genotype to his or her likelihood of crossing meaningful educational thresholds.

EDUCATIONAL outcomes are “heritable”; they tend to be more similar among more genetically similar individuals (for example, siblings as compared to cousins; Branigan, McCallum, and Freese 2013; Polderman et al. 2015). Recently, genome-wide association study (GWAS) designs have been used to identify molecular genetic correlates of educational attainment (Lee et al. forthcoming; Okbay et al. 2016; Rietveld et al. 2013). GWAS results can then be used as a scoring algorithm to construct a polygenic score (PGS), a summary measurement quantifying genome-wide genetic influence on some target phenotype (Dudbridge 2013). Critically, unlike heritability, a PGS is an individual-level measurement that can be calculated for a person from his or her DNA, allowing social scientists to integrate genetics into standard biosocial models of behavior. A polygenic score constructed based on results from a recent educational attainment GWAS explains more than 10 percent of observed variation in educational attainment (Lee et al. forthcoming) and has been replicated in multiple samples spanning several continents (Belsky et al. 2016; Okbay et al. 2016; Rietveld et al. 2013, 2014). Further, within-sibling analyses, which use family fixed effects to isolate the effects of genetic differences from the effects of environmental differences, suggest that the educational attainment PGS does so largely by indexing genetic differences that play causal roles (Belsky et al. 2018; Domingue et al. 2015).

Although there is substantial evidence linking the educational attainment PGS directly to educational attainment, we are only beginning to understand how this relationship is situated within broader educational attainment processes. Educational attainments vary substantially across schools, and there is evidence that some of this variation reflects the causal effects of school characteristics on students’ educational outcomes (Chetty et al. 2011; Chetty, Friedman, and Rockoff 2011). Yet, we know little about the degree to which genetic influences on educational and occupational attainment may be moderated by such environmental forces. For example, some analyses of heritability suggest that genetic differences may be more influential in higher-socioeconomic status (SES) environments (Tucker-Drob and Bates 2015; Turkheimer et al. 2003). However, not all evidence suggests the same conclusion (Figlio et al. 2017). Given the heterogeneity across school environments in the United States, understanding the degree to which there is interplay between one’s educational environment and genotype will inform our interpretation of predictions using the educational attainment PGS.

Educational attainment is a critically important social outcome, but it is an incomplete characterization of social position (Mood 2017); other life course attainments are also relevant. In particular, occupation is key to understanding processes of social attainment (Jonsson et al. 2009). Although the GWAS results we study here were trained to predict educational attainment, they also predict a broader set of socioeconomic attainments net of educational attainment, including occupational attainment (Belsky et al. 2016; Papageorge and Thom 2016). Thus, we additionally consider heterogeneity in the association between individual genotype and educational environment when predicting occupational attainment. We also consider the moderation of the relationship between an individual’s polygenic score and his or her probability of crossing specific educational thresholds (i.e., credentialing). We specifically focus on enrollment in and completion of postsecondary education.

To explore how the relationship between genotype and educational attainment may vary across contexts, we use data from two biosocial longitudinal studies that followed students from secondary school through mid- to late life. We test for school-level moderation of the association between the educational attainment PGS and both educational and occupational attainments. To contextualize findings, we conduct parallel analyses of two established predictors of educational attainment: family socioeconomic status and cognitive ability. Our analyses suggest that, although the educational attainment PGS is a robust predictor of individual outcomes, school-level environmental moderation of the educational attainment PGS’s association with educational and occupational attainments is likely to be small. Nonetheless, the probability that a person with a given value regarding his or her educational attainment PGS will make important educational transitions (to postsecondary education and subsequent college completion) is moderated by school-level factors, such as school socioeconomic status. However, this moderation is driven by changes in the expected outcome for a given PGS across social contexts (i.e., horizontal shifts in the distribution) and is not due to a change in the linear association between the PGS and outcome across context. These findings replicate across multiple data sets and methodologies.

## School-Level Moderation of Genetic Effects

A gene-environment interaction (GxE) is the existence of heterogeneous genetic effects across different environmental conditions. Knowledge of GxE is important for interpreting genetic effects; when GxE exists, efforts to explore how genotype influences phenotype must be contextualized. As the intersection of individual-level differences and group-level social structures and processes, questions regarding GxE attract substantial interest from social scientists studying the genetic influences of social outcomes.

In the following section, we discuss our framework for studying GxE. Before doing so, we discuss two methodological challenges relevant to such a pursuit. One challenge that has historically plagued the GxE literature is a lack of statistical power (Culverhouse et al. 2017; Duncan and Keller 2011). Our study uses polygenic score methods to combine information from education-linked genetic loci across the genome, yielding a relatively strong genetic predictor. A second challenge is the potential endogeneity of environmental exposures to genotypes (Fletcher and Conley 2013). Such endogeneity may arise from gene-environment correlations (rGEs), in which environmental exposures are associated with one’s genotype (Kendler and Baker 2007; Krapohl et al. 2017; Plomin and Bergeman 1991). As a consequence, we limit our inquiry to the investigation of the potential moderation of the statistical association between genotype and attainment outcomes, leaving any causal claims for future research.

### Our Framework for Examining GxE

Previous GxE work has a poor replication record (Duncan and Keller 2011; Young-Wolff, Enoch, and Prescott 2011), which has led to skepticism about this line of inquiry (Eaves 2006; Munafò and Flint 2009). Further, as the focal estimand is typically an interaction term, results from GxE studies are known to be sensitive to model specification decisions (Keller 2014; Tchetgen and Kraft 2011). Given these previous problems, we attempt to be precise about the specific data-generating mechanisms we envision giving rise to GxE (see Section A of the online supplement) as well as our power for identifying GxE under different scenarios.

The primary challenge is that we lack knowledge of which, if any, school-level environmental variables may be moderating the relationship between the PGS and educational attainment. To circumvent this problem, we first consider an “indirect” model of GxE, wherein we only examine variation in the correlation between the outcome and PGS across schools (remaining agnostic about the specific environment that may be driving this variation). We then conduct specific tests of GxE based on two candidate environmental variables that have been shown to be of interest in previous work (which we discuss in the next section).

### School Environments and GxE

The effects of schools on student attainments are a core interest of educational research. Studies dating back to at least the Coleman report (1968) document how school characteristics influence students’ future educational, social, and economic outcomes (Barnard 2004; Fonagy et al. 2005; Fuller and Clarke 1994). For example, school SES is a reliable correlate of student attainments (Perry and McConney 2010). GxE, however, requires the identification of subtler environmental influences (Boardman, Daw, and Freese 2013). In the traditional study of GxE, the key question is not which environments influence student attainments (we have knowledge of numerous environments that do so). Rather, the challenge has been to identify environments that restructure the relationship between genotype and educational attainment.

It is not immediately obvious which measurable school-level features may moderate the association between genotype and educational attainment in this way. Moreover, many measurable school environments may simultaneously contribute to moderation; for example, theoretical literature suggests that the effects of the genome on life course attainments may depend on both resource inequality and social mobility (Adkins and Vaisey 2009). We address this uncertainty by first studying variation in school-level associations between the educational attainment PGS and outcomes (i.e., equation 2 in the online supplement). Such an approach is agnostic as to which of the many school-level environments may matter and is analogous to school effect research that focuses on the existence of school-level variation in outcomes (Raudenbush and Willms 1995) rather than variation due to an identifiable school-level environment. In some circumstances, this strategy may have reduced statistical power. Thus, we also conduct tests related to two candidate environmental moderators (i.e., equation 3 in the online supplement).

The first candidate environment that we test is an overall measure of school socioeconomic status based on parents’ education. It has long been known that school SES is highly correlated with other indicators of overall school quality (Baker, Goesling, and LeTendre 2002; Hoover-Dempsey, Bassler, and Brissie 1987). We anticipate that this environmental measure will be strongly associated with both educational and occupational attainments of respondents, but it is not clear a priori that we should expect school SES to moderate the returns to an individual’s education-related genotype. The second candidate environment is a measure of school stratification. We consider a measure of inequality in parents’ education, hypothesizing that schools with high levels of inequality in parental education may be more rigidly stratified (e.g., may be more likely to “track” students). In earlier work (Boardman, Domingue, and Fletcher 2012), this school environment appeared to moderate the degree to which friends were genetically similar. Although not exhaustive of all environments that one might wish to measure at the school level, these candidate environments are reflective of the types of environments one might use in GxE studies.

## Methods

### Data

We deploy two data sets in studying whether schools moderate the influence of the educational attainment PGS: The Wisconsin Longitudinal Study (WLS) (Herd, Carr, and Roan 2014) and The National Longitudinal Study of Adolescent to Adult Heath (Add Health) (Harris 2013; Harris et al. 2013). Following the assay of biospecimens, genome-wide data are available for approximately 9,100 WLS members and for approximately 9,500 Add Health members. PGS analysis in diverse samples is currently not feasible (Martin et al. 2017), so we focus on subsamples of respondents of European ancestry given that such samples were the training data in the original GWAS. There exists a paucity of longitudinal data sources that have both molecular genetic data and clusters of respondents in common schools. We use the innovative approach of combining the WLS (N = 8,494) and Add Health (N = 4,915) to study the interaction of school environments and individual genotypes. Additional details on these data are available in Section B1 of the online supplement.

### Measures

We briefly describe the key measures used in this study here. Additional details on their construction and characteristics are included in Section B2 of the online supplement.

#### Outcomes.

Given that the educational attainment PGS is associated with a variety of life course attainments (Belsky et al. 2016; Papageorge and Thom 2016), we consider the outcomes related to time in school and job status. We consider educational attainment, which is measured as the years of completed education when the Add Health respondents were aged 24 to 32 and the WLS respondents were in their mid-40s. We also consider indicators of whether they engaged in any postsecondary schooling (>12 years of education) and were college graduates (≥16 years of education). Finally, we consider a measure of job status (Hauser and Warren 1997) based on jobs reported by respondents in 2008 for Add Health and 1992 for the WLS.

#### Predictors.

We focus primarily on a polygenic score constructed to predict educational attainment based on the most recently available GWAS for this phenotype (Lee et al. forthcoming). Alongside the educational attainment PGS, we examine household socioeconomic status and early-life cognitive functioning as additional individual-level measures related to life course attainments. We use results from these analyses as benchmarks for evaluating the magnitude of the relationships observed with the PGS.

#### Candidate environments.

We consider two candidate school environments. The first, the mean percentage of mothers with at least a high school diploma, is meant to represent school status. The second, the Gini coefficient in reported levels of parental education, is meant to represent school stratification. In both data sets, we construct these measures for those schools with at least 10 students for whom we have data. We interpret our measures as noisy proxies for the true environments of interest and explore the consequences of this possible measurement error in our power analysis.

### Analysis

As discussed in the section on school environments and GxE, we first examine the possibility of school-level GxE using an approach that is agnostic as to which specific feature of the environment may be relevant. We do this via the estimation of a random effects model; in particular, we examine a model of the form (where individual *i* is in school *j*):

(1) $$\text{Indirect}\;:\;{\text{Outcome}}_{ij}=\beta_{0}+\mu_{j}+(\beta_{1}+\delta_{j})\;{\text{PGS}}_{ij}+X^{\prime }\beta +\epsilon_{ij}.$$

We additionally assume that (*μ_j_*, *δ_j_*) ~ multivariate normal[0,Ω]. The focal parameter here will be the variation in *δ_j_* as captured by estimates of the covariance matrix Ω (i.e., ${\hat{\sigma }}_{\delta }$). To the extent that estimates of this quantity are near zero, this suggests that the effect of the PGS is relatively constant across all schools observed in our data. We also consider a modified version of equation 1 wherein we first mean center years of education (or other outcomes) in each school and thus do not include the random intercept term *μ_j_*. In all analyses, the focal predictors and outcomes are standardized, and we include sex and birth year as covariates. For the WLS, we also include a family-specific random effect to account for sibling relatedness.

Evaluations of nearness to zero as they pertain to estimates of variance components, such as ${\hat{\sigma }}_{\delta }$, need to be made carefully. To aid our interpretations of estimates of equation 1, we rely upon a variation of a Fisher exact test (Athey and Imbens 2017) wherein respondents are randomly assigned to the set of schools in the data (i.e., we ignore actual school assignment). A Fisher exact test is a form of randomization inference that involves comparing an observed distribution of outcomes to many simulated distributions under a null hypothesis; here, the null hypothesis is that there is no school-level moderation of the educational attainment PGS. Utilizing such a test allows for the detection of statistically significant variation of association between the PGS and educational attainment at the school level. We evaluate the magnitude of our observed $\hat{\Omega }$ relative to the distribution of simulated ${\hat{\Omega }}_{r}$. Specifically, we focus on the quantile rank of elements of $\hat{\Omega }$ relative to the distribution of ${\hat{\Omega }}_{r}$ after repeated randomizations. We examine one minus the mean quantile rank, which we treat as a *p* value for the implied randomization test.

We then turn to analyses that focus on two candidate environments: school status and school stratification. For analyses based on the these candidate school environments *E_j_*, we estimate the following:

(2) $$\text{Direct}\;:\;{\text{Outcome}}_{ij}=\beta_{0}+u_{j}+\beta_{1}{\text{PGS}}_{ij}+\beta_{2}E_{j}+\beta_{3}{\text{PGS}}_{ij}E_{j}+X^{\prime }\beta +\epsilon_{ij}.$$

Note that we allow differences in mean school outcomes via the inclusion of the random effect *u_j_*. Interest resides in estimates of *β*_3_. To guard against spurious findings of GxE (Keller 2014), the *X*′ *β* term also includes interactions between the key predictors (PGS*_ij_* and *E_j_*) and other control variables (sex and birth year).

## Results

### Gene–Environment Correlation

**Figure 1:**
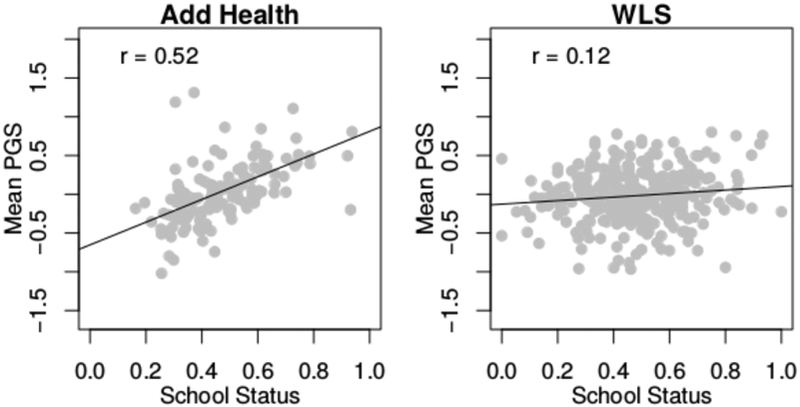
Mean PGS for the respondent as a function of school status (percent of parents with a high school diploma) in each data set. PGS, polygenic score; WLS, Wisconsin Longitudinal Study.

The school a child attends is not independent of genotype; subsequent findings will need to be interpreted in light of this selection process. Specifically, 6.5 percent of the variation in the educational attainment PGS is between schools in Add Health compared to 2.1 percent in the WLS (see Table S1 in online supplement). These findings are consistent with those previously observed between school types in the United Kingdom (Smith-Woolley et al. 2018). However, the educational attainment PGS is clustered within schools to a much lesser degree than other individual-level predictors. Approximately 17 to 27 percent of the household SES and 7 to 10 percent of the cognitive functioning variation is between schools. One consequence of this clustering is that we observe an association between the school-mean educational attainment PGS and school status. Figure 1 shows the school-mean educational attainment PGS as a function of our environmental measure related to school status (i.e., the proportion of mothers who finish high school in the school). In Add Health, these figures are highly correlated (r = 0.52), but even in the WLS, there is an observable gradient (r = 0.12). To address this potential source of bias, we focus interpretation on a model in which the outcome is school centered in our indirect GxE analyses.

### Power Analysis

**Figure 2:**
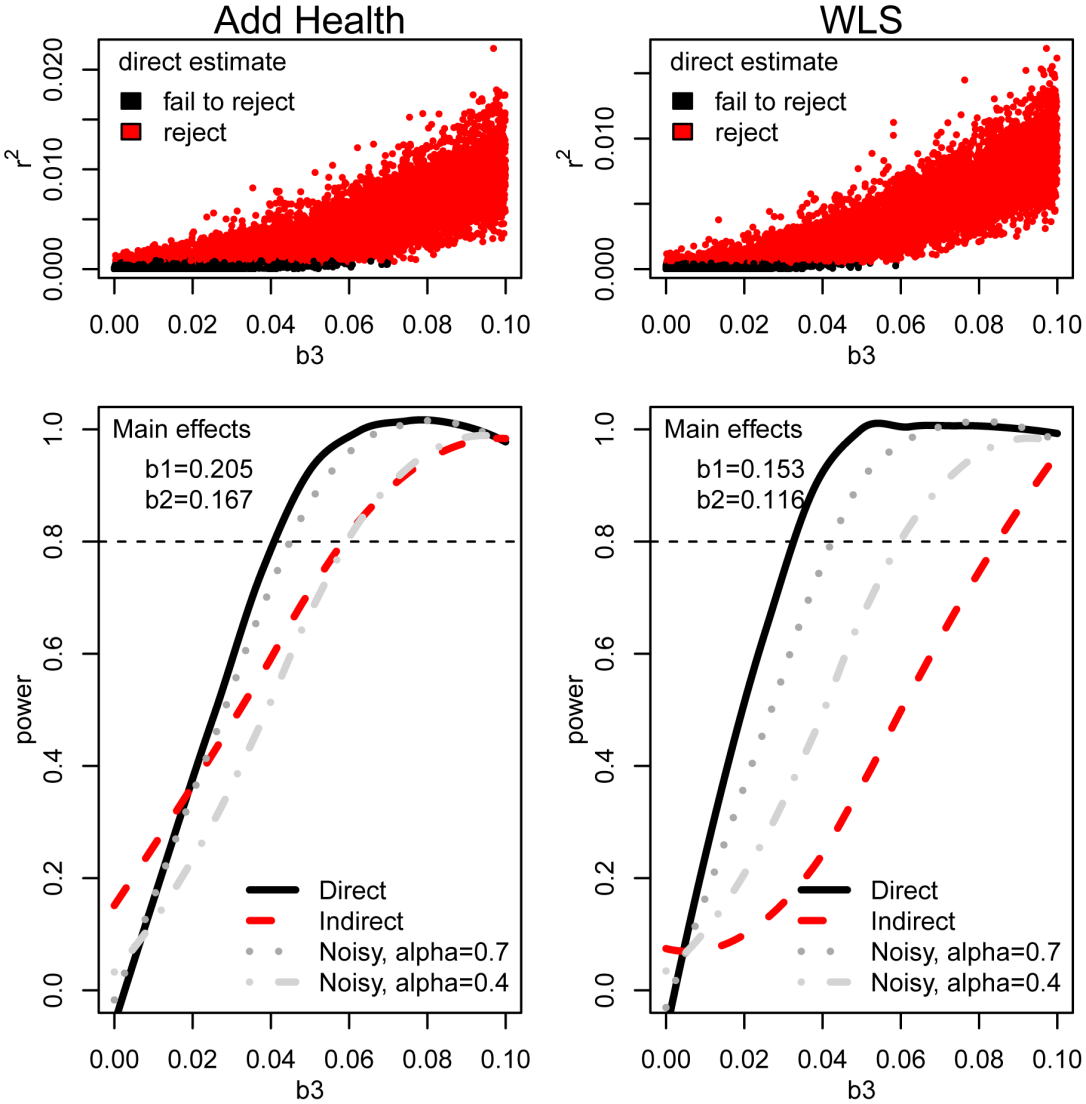
Power analysis based on 10,000 random GxE coefficients (b3; x axis) and specified main effect values (b1 = genetic main effect; b2 = environment main effect). WLS, Wisconsin Longitudinal Study.

Power curves for the detection of a single environmental moderator are shown in Figure 2 (see details in Section C of the online supplement). If we observe the environmental moderator without error (black line), then we have sufficient power to detect interactions using the direct approach when the interaction coefficient is approximately one-fifth the size of the main genetic effect (power of 0.8 is obtained when interaction coefficients are around 0.04 in Add Health and 0.03 in the WLS). Note that these correspond to small amounts of additional explained variance; observed r^2^ values are less than 0.005 (see top panels in Figure 2). Our indirect approach (red line) has less power; we can only detect interactions that are roughly twice as large as in the direct approach. We also consider power based on noisy observations of the environmental moderator; here, direct analysis based on an environmental moderator measured with a great deal of noise (e.g., *α* = 0.4) still offers superior power to the indirect approach in the WLS but not in Add Health.

### Evidence from Indirect Analyses

#### Educational attainment.

**Figure 3:**
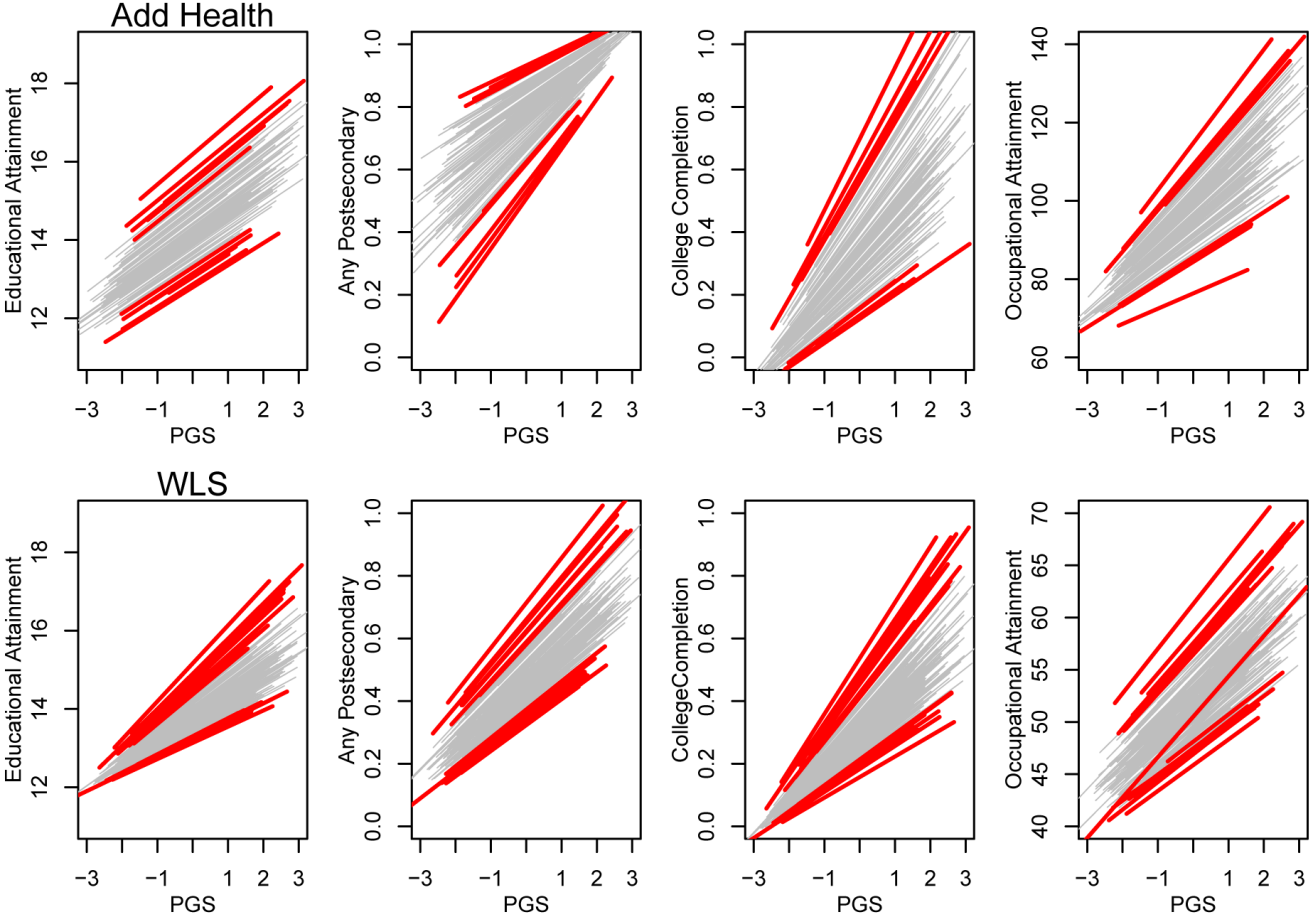
Prototypical plots for outcomes as a function of educational attainment PGS (for those schools with at least 10 respondents). Each line represents a school-level association between the PGS and the relevant outcome. Thick, red lines show the schools with slopes in the top and/or bottom 5 percent of the distribution of slopes in each panel. PGS, polygenic score; WLS, Wisconsin Longitudinal Study.

Focusing first on the models with random intercepts, increases in the educational attainment PGS are associated with additional educational attainment (in standardized units, b = 0.31 in Add Health and b = 0.24 in the WLS [see left half of Table 1]; in raw years of educational attainment, 0.67 years in Add Health and 0.55 years in the WLS). The estimated SD of slopes, ${\hat{\sigma }}_{\delta }$, is 0.033 in Add Health and 0.060 in the WLS. Illustrations of this type of variation can be found in Figure 3; the increased variation in slopes in the WLS relative to Add Health is apparent. In particular, note that there is a concentration of all trajectories around 12 years of schooling in the WLS. As we discuss later, this is largely because most of the respondents in the WLS had to complete 12 years of education to be eligible for the study.

In Add Health, the magnitudes of the variance components related to the educational attainment PGS are largely consistent with estimates derived from randomization analyses in which there is no school-level moderation of the PGS’s effect (*p* = 0.199; see Table 1). In the WLS, there is some weak initial evidence for moderation (*p* = 0.048). However, we interpret this result cautiously. There is a strong estimated correlation ($\hat{\sigma_{\mu \delta }}$) between the slopes and intercepts, suggesting that the effect of the PGS on educational attainment is highest in schools with the highest average levels of educational attainment. In contrast to what we observe with respect to variation in the slopes, we observe large variation in the intercepts, as measured by $\hat{\sigma_{\mu }}$, in both data sets. All data sets with students randomly assigned to schools produce much smaller variation in the intercepts than what is estimated in either empirical data set; we elaborate on this point below.

To further examine the implications of the strong correlation between random slopes and intercepts, we estimate a version of equation 1 that does not include random intercepts using data for which the outcome is centered within the school. Estimates of the effect of the PGS are similar to those noted above (b = 0.28 in Add Health and b = 0.22 in the WLS; see right half of Table 1 as well as Table S2 in the online supplement). The estimated variation in slopes, ${\hat{\sigma }}_{\delta }$, is now larger in Add Health than in the WLS. In both data sets, we observe randomization *p* values associated with this quantity around 0.05 (*p* = 0.06 in Add Health; *p* = 0.04 in the WLS).

We also examine variation in associations between our two outcomes related to academic thresholds—any postsecondary education and college completion—and the educational attainment PGS. For Add Health, there is more variation in the association between the educational attainment PGS and college completion than in the randomization data sets (*p* = 0.02 in the mean-centered analysis). In contrast, for the WLS, there is more variation in the slopes for the any-postsecondary analysis (*p* = 0.02 in the mean-centered analysis).

#### Job status.

The educational attainment PGS is robustly associated with job status in both data sets (see Table 1). Note that the distributions of job status (see Figure S1 in the online supplement) are more approximately normal than the distributions for educational attainment. In comparison to the educational attainment results in Add Health (where all the lines in Figure 3 are relatively parallel), there is potentially more variation in the association between the educational attainment PGS and job status. However, focusing on the mean-centered analyses, there is no evidence for a substantial difference in association between the PGS and job status across schools in either the Add Health (*p* = 0.16) or the WLS (*p* = 0.10).

#### Alternative predictors.

To better contextualize our findings related to the educational attainment PGS, we also consider results based on replacing the educational attainment PGS with either a measure of cognition or childhood socioeconomic status. As expected, these quantities are strong predictors of both attainment measures: educational achievement and job status (see Table S1 in the online supplement; note that the educational attainment PGS correlates with educational attainment at 0.26–0.36, whereas the other predictors show correlations with educational attainment above 0.37). In contrast to the results based on the educational attainment PGS, there is strong evidence for school-level moderation of both the SES and cognition link to educational attainment in both data sets (Table 1). There is also evidence of school-level moderation of the association between household SES and occupational returns. Note both the strength and consistency of these findings. In comparison to these findings, the previously discussed evidence for the moderation of genetic effects is relatively weak.

#### Variation in returns.

**Figure 4:**
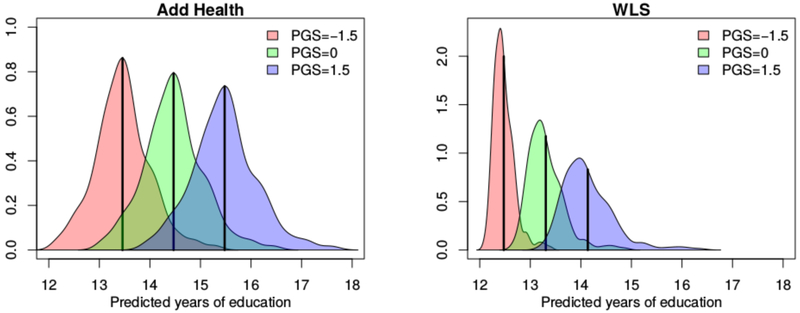
Distribution of predicted years of education across schools for fixed values of the educational attainment polygenic score (PGS). WLS, Wisconsin Longitudinal Study.

An obvious implication of the results described in Table 1 pertains to the substantial variation in returns to a given genotype as a function of school assignment (i.e., the variation in intercepts, $\hat{\sigma_{\mu }}$). There is far more variation in the intercepts in our empirical data than in the data sets in which the school is randomly assigned. Figure 4 illustrates this fact by showing the distribution of predicted years of educational attainment across schools for three different values of the PGS. Consider the results in Add Health. For the mean PGS (the green distribution), there is more than two years of variation in the expected educational attainment of genetically similar respondents as a function of attending different schools. Students with the mean educational attainment PGS would be expected to get around 13 years of schooling if they attend some schools and 15 or more years of schooling if they attend other schools. The difference is pronounced and consistent with previous reports of substantial differences in school quality and educational opportunity (Card and Krueger 1992). Irrespective of students’ genotypes, the school environment is strongly associated with how far a student will go in school. Results in the WLS are slightly different. At the low end of the PGS distribution, we see relatively tight clustering around 12 to 13 years of schooling. At the high end, however, there is increased variation in the potential outcomes.

### Direct Analyses

**Figure 5:**
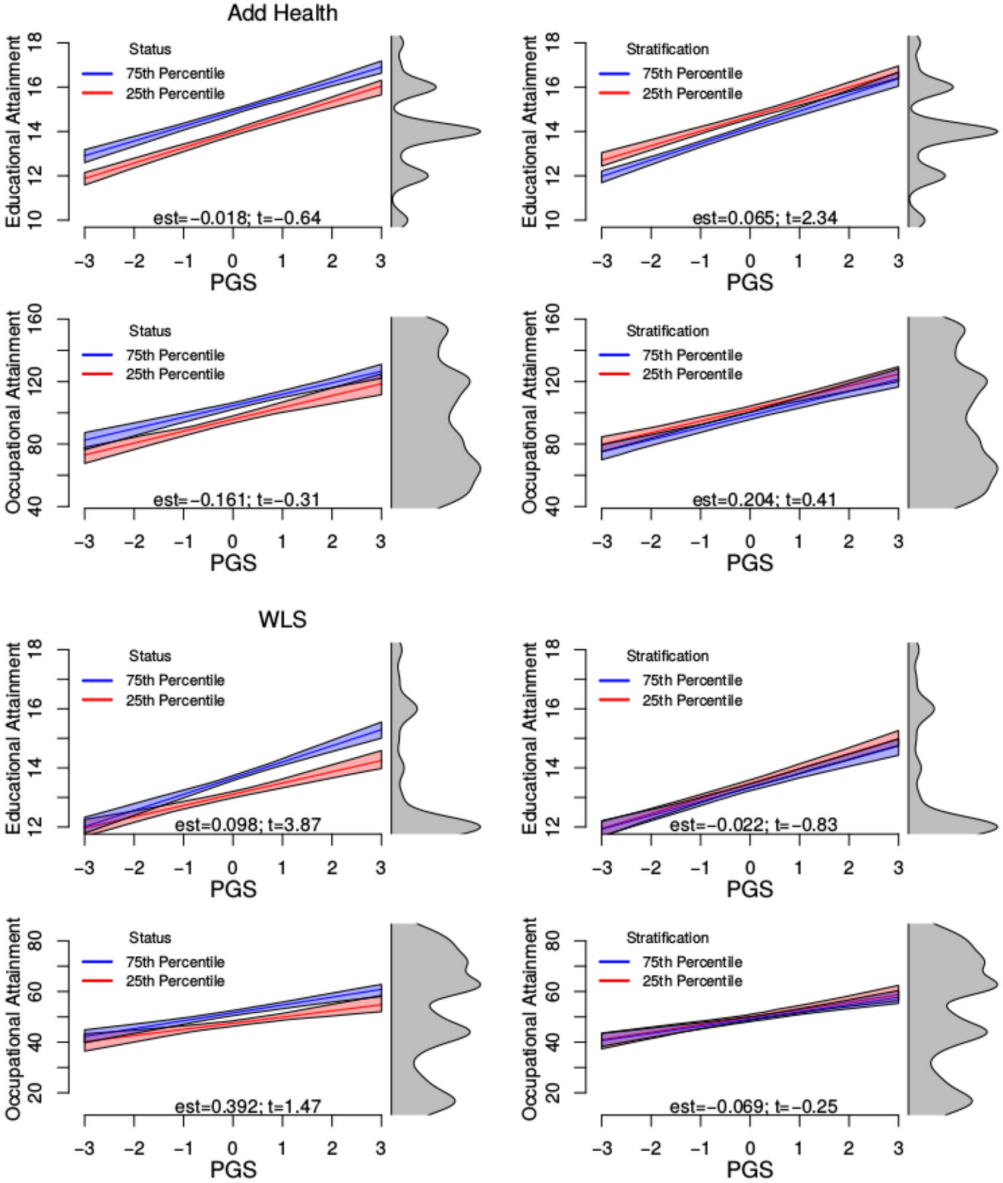
Prototypical plots for direct tests of GxE with candidate environments. Environments are at the 25th and 75th percentiles of the school-level distribution. Results are for females of mean age (in the WLS, they are also assumed to be graduate respondents). The right-hand side of each panel shows the distribution of the variable on the y axis. est, estimate; PGS, polygenic score; WLS, Wisconsin Longitudinal Study.

Evidence is mixed regarding the school-level environmental moderation of the association between the PGS and educational attainment by school status and stratification (Table 2). In Add Health, the gradient between years of schooling and the educational attainment PGS is steeper in more **stratified** schools. In contrast, in the WLS, this gradient is steeper in higher-*status* schools (Figure 5; note that results in these figures are not based on a standardized outcome so as to aid interpretation). We interpret these results as weak signals of school moderation for several reasons. First, findings do not replicate across data sets. This may be due to structural changes (i.e., period-related differences in schools) that exist between the educational systems encountered by the WLS respondents as compared to the Add Health respondents (we also explore the role of the inclusion of graduate and sibling respondents in the WLS; see Section D of the online supplement). Furthermore, findings regarding the moderation of associations between the educational attainment PGS and educational attainment do not translate into moderations of associations between the educational attainment PGS and downstream occupational attainments. Finally, these results are not robustly foreshadowed by the indirect analyses in the section of the same title.

**Figure 6:**
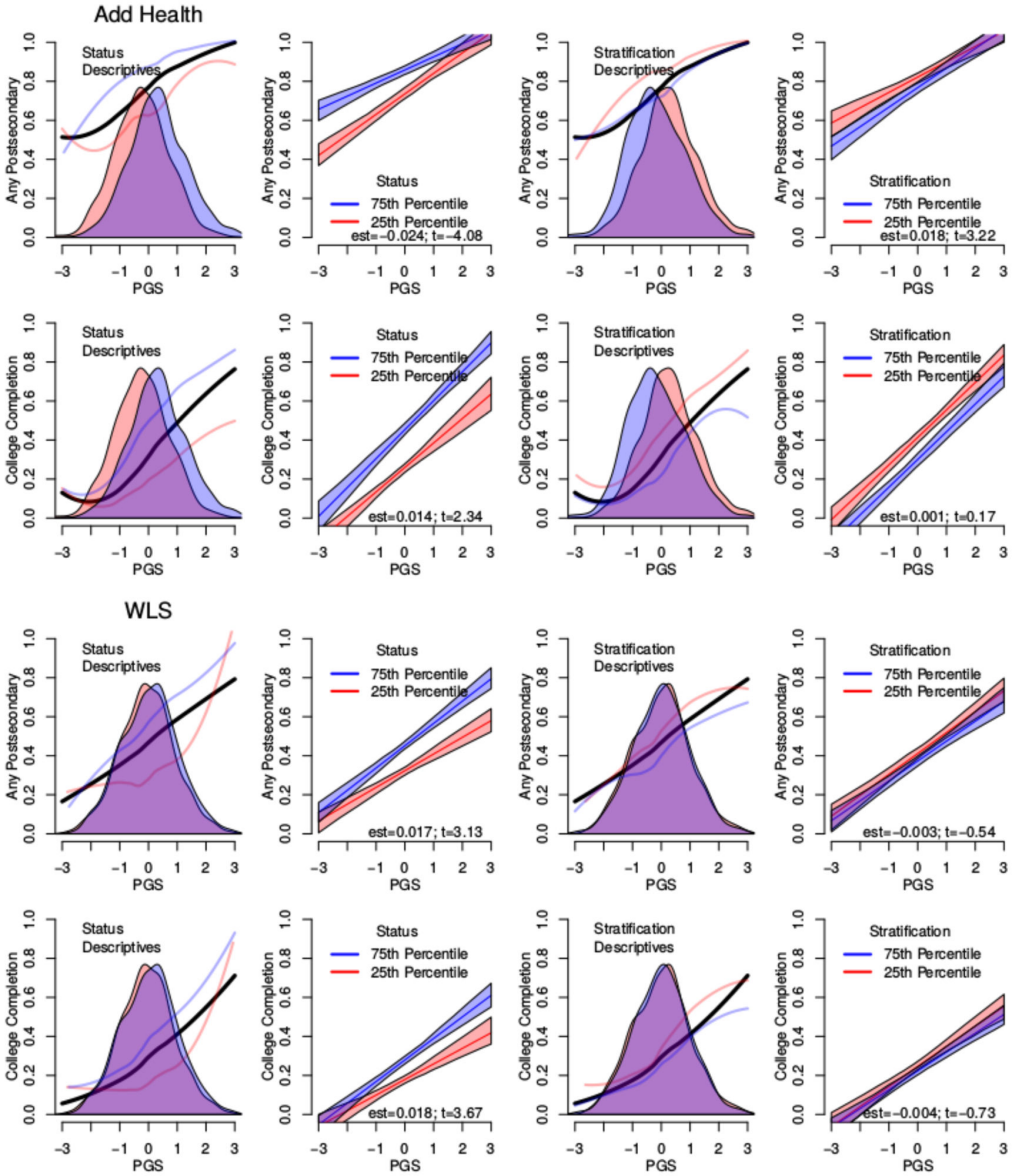
Prototypical plots for direct tests of GxE with candidate environments. Environments are at the 25th and 75th percentiles of the school-level distribution. Results are for females of a mean age (in the WLS, they are also assumed to be graduate respondents). Descriptive panels show distributions (shaded) for students in schools below the 25th percentile and above the 75th percentile (red and blue, respectively). LOESS curves plot fitted probabilities as a function of the PGS for all respondents (black) and those in schools captured in the density plot of same color. est, estimate; PGS, polygenic score; WLS, Wisconsin Longitudinal Study.

Results based on crossing educational thresholds are more intriguing. We focus on any postsecondary education (more than 12 years of schooling) and the acquisition of a college degree (16 or more years of schooling). As anticipated by Table 1, Table 2 suggests that genetic associations with both postsecondary enrollment and college completion may be moderated by school status. We focus on these results in Figure 6. To enhance the results from linear probability models, we also include panels emphasizing descriptive analysis. In these panels, we show distributions of polygenic scores for those students in the top and bottom quartiles of schools in the respective distributions (for consideration of rGE; note that PGS distributions in high- and low-status schools are more comparable in the WLS than Add Health) as well as a locally weighted scatterplot smoothing (LOESS)-fitted line describing associations between the polygenic score and the probability of either outcome for students in the different schools. These nonlinear trends can then be compared to the linear fits.

School status clearly moderates the probability of postsecondary schooling or college completion in both data sets. Consider first enrollment in postsecondary schooling. In the WLS, the interaction is positive. That is, students from higher-status schools were increasingly more likely to enroll in postsecondary education as their PGSs increased. In contrast, the interaction coefficient in Add Health is negative. This is due to the fact that most students in high-status schools from that cohort are already attending some postsecondary school; there is a limited role for genetics to play For both the WLS and Add Health study members, the association between one’s genetics and completing college were more pronounced for students who attended higher-status schools.

Turning to the other predictors (social origin and cognitive functioning), the association of social origin with educational attainment appears to be consistent across these two environments. In contrast, the effect of cognitive functioning on educational attainment seems to be moderated by school status in both data sets. However, this moderation is of an inconsistent sign; further work is perhaps needed to identify whether certain measurable school-level environments are reliable moderators of these individual-level variables.

## Discussion

Findings from two longitudinal studies of those born in the United States roughly a half-century apart suggest that school-level moderation of genetic influences on educational attainment—as captured by a PGS constructed using the third-generation GWAS of this outcome (Lee et al. forthcoming)—are likely to be, in general, small. The PGS is a robust predictor of educational and occupational attainments whose predictive power may vary slightly across schools but does not seem to do so as a function of the measured environments we consider here. In contrast, we observe evidence for the school-level moderation of the relationship between individual cognitive functioning or SES and the related set of life course attainments (although it is sometimes unclear which specific school-level environments may lead to such moderation).

A key exception to the results described above is the moderation of the probability of the two binary outcomes related to postsecondary education for a given PGS. Our analyses for both any postsecondary education and college completion showed evidence of moderation by school SES. We observe (Figure 6) that at a time when higher education was less common (Bailey and Dynarski 2011)—that is, when the WLS respondents were young—higher educational attainment PGS–students from higher-status schools were much more likely to attend any postsecondary schooling. In contrast, for those who attended school more recently (i.e., the Add Health respondents), enrollment in any postsecondary schooling was more common and even low-PGS students at high-status schools are likely to be in higher education, resulting in a relatively flat slope for these schools. The inconsistency of sign for the GxE coefficient estimates for postsecondary enrollment is interpretable in light of Figure 4. The amount of education predicted by an individual’s educational attainment PGS varies substantially across the two study periods (an Add Health respondent with a mean PGS would expect to get 14–15 years of schooling, whereas a WLS respondent with a mean PGS would expect closer to 13 years of schooling; see Figure 4). The changing baseline rate of college attendance mechanically alters the relationship between genes and the environment.

This finding emphasizes important differences between these two studies that make generalization challenging. The WLS is a more homogeneous cohort from an earlier historical period wherein students tended to be in school for fewer years overall; more students dropped out of high school, and fewer received postsecondary education (Heckman and LaFontaine 2010). Moreover, WLS respondents were genotyped later in life, so mortality selection may also complicate these findings (Domingue et al. 2017). Finally, respondents in the WLS have a truncated distribution of educational attainment (see Figure S1 in the online supplement), as participants of the WLS had to graduate high school to be eligible to be empaneled (or be a sibling of such a graduate).

There is also the potential for endogeneity that complicates the interpretation of our findings. For example, the mean status of the school that a student attends is correlated with the school’s mean polygenic score (see Figure 1). Similarly, endogeneity also exists with respect to the results focusing on SES and cognitive functioning as these are also associated with school choice and potentially associated with individual genotype. With respect to SES, the childhood socioeconomic environment is associated with both an individual’s genetics (Belsky et al. 2016) as well as the genetics of the parents (Belsky et al. 2018; Conley et al. 2015). Indeed, recent results suggest that parental genetics may have implications for offspring even when not directly transmitted (Kong et al. 2018). Cognitive functioning as measured in either study is almost surely related to the educational environments to which the respondent was exposed up until that point in the life course. In general, genotypes are also not randomly distributed across environments (Domingue et al. 2018a; Domingue et al. 2018b; Haworth et al. 2018), making the interpretation of GxE research challenging.

Despite these limitations, our findings demonstrate several important points. The difference in the distribution of educational attainment across the two data sets highlights an important fact about the interpretation of results from GxE studies. The identification of GxE offers crucial guidance for the interpretation of genetic effects but is not necessarily informative about the underlying cause of the observed GxE. For example, the observed moderation might be due to environmental constraints placed on the variation in phenotype (Tropf et al. 2017) along the lines observed here. On the other hand, we might observe moderation due to the fact that a genetic variant has effects in opposite “directions” across contexts (this issue relates to the distinction between stress-diathesis versus differential susceptibility models; Ellis et al. 2011).

When a genetic variant does, in fact, have effects in opposite directions across contexts, the notion of “genetic risk” is environmentally contingent. However, as we discuss below, results based on polygenic scores are unlikely to capture this type of environmental contingency. Instead, more attention should be paid to how an environment constricts or expands the distribution of the phenotype in question. For example, a constant PGS computed based on a GWAS for body mass index (BMI) predicts a larger BMI for someone born now rather than in the past (Conley et al. 2016; Liu and Guo 2015; Walter et al. 2016). An increase in the PGS for BMI consistently predicts increased BMI; it is just that the BMI distribution has changed over these birth cohorts (Kuczmarski et al. 1994). In rank-order terms, it is certainly not the case that the same genetic profile predicted a relatively slender person (as compared to peers from his or her birth cohort) born in 1950 and a relatively heavy person born in 1990. Such a finding would have profound implications; indeed, it would raise questions about the validity of the results obtained via GWAS.

Rather, our most interesting findings seem to hinge on observable changes in the distribution of the phenotype. Figures 3 and 4 show that there is effectively a floor in educational attainment for WLS participants; nearly all students get at least 12 years of education irrespective of their educational attainment PGS. In contrast, in some schools, the students with a higher educational attainment PGS go on to college, whereas in other schools, they do not. This mechanical constriction of variation at the low end of the attainment ladder may lead to the observed GxE in Table 1 for the PGS’s changing influence on attainment. We favor this structural interpretation given that the variation in association observed with respect to educational attainment does not translate into any such variation in the association with occupational attainment (see also Section D of online supplement). This finding in the WLS is similar to the recent observation that a similar polygenic score predicts additional variance in outcomes in Estonia in the post-Soviet period (Rimfeld et al. 2018) or to the reduction in health disparities linked to genotype after the introduction of a compulsory schooling law (Barcellos, Carvalho, and Turley 2018). These findings all tie reductions in phenotypic variance associated with specific contextual paradigms to reductions in associations with the relevant genetic predictor. Future work of GxE inquiry and interpretation may benefit from careful considerations of how the relevant phenotypic distributions vary across environments before genetic analyses are scrutinized.

Our findings are also worth interpreting in light of recent work discussing differences in academic achievement across school types and the potential role of genetics (Smith-Woolley et al. 2018). They show that differences in the distributions of polygenic scores between nonselective, grammar, and private schools explain some of the difference in academic achievement across the school types. This leads them to the conclusion that differences in the mean academic achievement of the three different school types are due in part to the differences in the underlying genetic composition of their students. However, the extent to which the returns to an individual’s polygenic score vary based on the environmental context is an important consideration in deciding whether findings entail this conclusion. In the data utilized here, there is some segregation of educational attainment PGS across schools, but an alternative hypothesis is available given the difference in expected returns, in the form of educational and occupational attainments, for a fixed educational attainment PGS across schools.

Our finding of a limited role for GxE in this context is perhaps unsurprising when one considers the methodology employed for the identification of the relevant genetic loci. GWAS is built to be a tool for the identification of single nucleotide polymorphisms (SNPs), whose variation is consistently associated with phenotypic variation. That is, polygenic scores are constructed based on SNPs that show the most reliable main effects and are thus less likely to be those loci that are particularly environmentally sensitive. If, for example, most GxE associations are those with effects whose signs vary as a function of environmental context—similar to a differential susceptibility model (Belsky and Pluess 2009) —then these SNPs are unlikely to be detected in a GWAS; simply differentiating loci that are true crossover SNPs from random variation is challenging (Boardman et al. 2014). Meta-analytic GWASs that combine data from a broad spectrum of places and time periods, as with the educational attainment GWAS, will identify only the genetic variants whose effects are robust to these environmental differences. Finally, because moderating environments may operate on a specific biological pathway, the act of summarizing thousands of different SNPs (that likely work through numerous biological pathways) into a single PGS complicates the detection of GxE.

We cannot rule out the moderation of all genetic effects on educational attainment, merely the ones emphasized in the educational attainment PGS studied here. Indeed, alternative methods based on genetic loci associated with variation in outcome (Conley et al. 2018) may provide different information about the consistency of genetic prediction across environments. Given the relevance of schools for life course attainments, this null finding provides important contextual information for the interpretation of current and forthcoming GWAS findings relating to educational attainment. Although we remain skeptical of the existence of substantial school-level moderation of the linear association between continuously measured attainments and the educational attainment PGS, our findings emphasize the importance of social context in the interpretation of genetic predictions. School context is crucial in connecting an individual’s genotype to his or her likelihood of crossing meaningful educational thresholds. Given our findings related to credentialing processes, conceptions of interplay between genes and environments should be expanded to include changes in levels associated with environmental context rather than simply changes in slopes.

Finally, it is important to recall that our results only apply to individuals with European ancestry in the two studies. We restricted our sample because differences in linkage disequilibrium and allele frequencies that exist across ancestral groups complicate the interpretation of PGS-phenotype associations (Martin et al. 2017). Although we recognize the importance of research in more diverse samples, our analysis is a first step in considering the role of the school environment in moderating the relationship between the educational attainment PGS and life course attainments.

## Supplementary Material

Supplemental Material

## Figures and Tables

**Table 1:** Comparison of estimated variance components from equation 1 (indirect approach) to those based on randomization analysis (as well as estimated coefficients for individual predictors). Results for polygenic score analyses are in bold. Randomization *p* values (italicized, along with SDs of slopes) are based on 1,000 random assignments of students to schools.

		Models with Random Intercept						Models with Mean Centering			
		
Outcome	Predictor	${\hat{\beta }}_{1}$	SE	$\hat{\sigma_{\mu }}$	PV	${\hat{\sigma }}_{\delta }$	*PV*	${\hat{\beta }}_{1}$	SE	${\hat{\sigma }}_{\delta }$	*PV*
Add Health											
**Education**	**PGS**	**0.312**	**0.013**	**0.323**	**0.000**	***0.033***	***0.199***	**0.275**	**0.014**	***0.057***	***0.060***
Education	SES	0.422	0.017	0.209	0.000	*0.095*	*0.004*	0.293	0.018	*0.131*	*0.000*
Education	COG	0.324	0.017	0.316	0.000	*0.107*	*0.000*	0.270	0.017	*0.113*	*0.000*
**Any Postsecondary**	**PGS**	**0.098**	**0.006**	**0.107**	**0.000**	***0.027***	***0.083***	**0.082**	**0.006**	***0.018***	***0.174***
Any Postsecondary	SES	0.138	0.007	0.063	0.000	*0.033*	*0.086*	0.091	0.007	*0.033*	*0.017*
Any Postsecondary	COG	0.111	0.008	0.101	0.000	*0.060*	*0.000*	0.090	0.008	*0.053*	*0.000*
**College Completion**	**PGS**	**0.134**	**0.007**	**0.133**	**0.000**	***0.035***	***0.003***	**0.117**	**0.007**	***0.026***	***0.021***
College Completion	SES	0.193	0.009	0.000	0.732	*0.063*	*0.000*	0.122	0.008	*0.054*	*0.000*
College Completion	COG	0.122	0.008	0.136	0.000	*0.039*	*0.005*	0.100	0.008	*0.047*	*0.000*
**Occupation**	**PGS**	**0.219**	**0.015**	**0.216**	**0.000**	***0.048***	***0.123***	**0.182**	**0.014**	***0.042***	***0.161***
Occupation	SES	0.330	0.015	0.000	0.667	*0.054*	*0.082*	0.209	0.016	*0.077*	*0.000*
Occupation	COG	0.236	0.014	0.213	0.000	*0.008*	*0.620*	0.194	0.014	*0.021*	*0.314*

WLS											
**Education**	**PGS**	**0.236**	**0.011**	**0.211**	**0.000**	***0.060***	***0.048***	**0.221**	**0.011**	***0.034***	***0.042***
Education	SES	0.373	0.012	0.114	0.000	*0.050*	*0.080*	0.262	0.014	*0.097*	*0.000*
Education	COG	0.407	0.012	0.184	0.000	*0.091*	*0.004*	0.358	0.012	*0.080*	*0.000*
**Any Postsecondary**	**PGS**	**0.103**	**0.005**	**0.109**	**0.000**	***0.016***	***0.051***	**0.096**	**0.006**	***0.020***	***0.021***
Any Postsecondary	SES	0.182	0.005	0.056	0.000	*0.002*	*0.418*	0.129	0.007	*0.042*	*0.000*
Any Postsecondary	COG	0.192	0.005	0.091	0.000	*0.018*	*0.001*	0.167	0.006	*0.037*	*0.000*
**College Completion**	**PGS**	**0.102**	**0.005**	**0.089**	**0.000**	***0.029***	***0.043***	**0.100**	**0.005**	***0.000***	***0.274***
College Completion	SES	0.156	0.006	0.055	0.000	*0.028*	*0.185*	0.114	0.006	*0.034*	*0.002*
College Completion	COG	0.177	0.006	0.074	0.000	*0.038*	*0.015*	0.156	0.005	*0.025*	*0.000*
**Occupation**	**PGS**	**0.142**	**0.013**	**0.200**	**0.000**	***0.035***	***0.139***	**0.130**	**0.012**	***0.034***	***0.097***
Occupation	SES	0.269	0.012	0.109	0.001	*0.034*	*0.173*	0.178	0.013	*0.050*	*0.000*
Occupation	COG	0.354	0.011	0.127	0.000	*0.007*	*0.531*	0.304	0.011	*0.013*	*0.176*

*Note*: COG, cognition; PGS, polygenic score; PV, *p* value ; SES, socioeconomic status; WLS, Wisconsin Longitudinal Study.

**Table 2:** Estimated GxE coefficients from equation 2 (direct approach) based on candidate environments (outcome and key predictors are standardized in all analyses).

			Add Health			WLS		
			
Outcome	Individual	Environment	*β*_3_	SE	PV	*β*_3_	SE	PV
Education	PGS	Status	−0.008	0.013	0.523	0.041	0.011	0.000
Education	PGS	Stratification	0.030	0.013	0.019	−0.009	0.011	0.407
Any Postsecondary	PGS	Status	−0.056	0.014	0.000	0.034	0.011	0.002
Any Postsecondary	PGS	Stratification	0.043	0.014	0.001	−0.006	0.011	0.590
College Completion	PGS	Status	0.031	0.013	0.019	0.040	0.011	0.000
College Completion	PGS	Stratification	0.002	0.013	0.867	−0.008	0.011	0.468
Occupation	PGS	Status	−0.004	0.014	0.753	0.017	0.012	0.141
Occupation	PGS	Stratification	0.005	0.013	0.684	−0.003	0.012	0.801
Education	SES	Status	0.023	0.014	0.111	0.016	0.010	0.103
Education	SES	Stratification	−0.006	0.015	0.665	−0.002	0.011	0.861
Any Postsecondary	SES	Status	−0.042	0.015	0.004	0.004	0.010	0.701
Any Postsecondary	SES	Stratification	0.023	0.015	0.122	0.005	0.011	0.643
College Completion	SES	Status	0.070	0.015	0.000	0.021	0.010	0.044
College Completion	SES	Stratification	−0.039	0.015	0.007	−0.008	0.011	0.495
Occupation	SES	Status	0.025	0.015	0.089	−0.021	0.011	0.054
Occupation	SES	Stratification	−0.020	0.015	0.188	0.013	0.012	0.268
Education	Cognition	Status	−0.044	0.014	0.002	0.037	0.010	0.000
Education	Cognition	Stratification	0.031	0.014	0.030	−0.002	0.010	0.807
Any Postsecondary	Cognition	Status	−0.087	0.014	0.000	0.017	0.010	0.090
Any Postsecondary	Cognition	Stratification	0.030	0.015	0.042	0.009	0.010	0.366
College Completion	Cognition	Status	0.006	0.014	0.682	0.036	0.010	0.000
College Completion	Cognition	Stratification	0.011	0.015	0.440	0.000	0.010	0.982
Occupation	Cognition	Status	−0.023	0.015	0.111	−0.008	0.011	0.467
Occupation	Cognition	Stratification	0.031	0.015	0.039	0.016	0.011	0.151

*Note:* PGS, polygenic score; PV, *p* value; SES, socioeconomic status; WLS, Wisconsin Longitudinal Study.
