# Supplementary material for: Schools as Moderators of Genetic Associations with Life Course Attainments: Evidence from the WLS and Add Health
Source: Sociol Sci. Author manuscript; Available in PMC 2019 Jan 2. (PMC6314676; doi:10.15195/v5.a22)

sociological science

Supplement to:

Trejo, Sam, Daniel W. Belsky, Jason D. Boardman, Jeremy Freese, Kathleen Mullan Harris, Pam Herd, Kamil Sicinski, and Benjamin W. Domingue. 2018. "Schools as Moderators of Genetic Associations with Life Course Attainments: Evidence from the WLS and Add Heath." Sociological Science 5: 513-540.

Schools as moderators of genetic associations with lifecourse attainments:
Evidence from the WLS and Add Heath

Online Supplement

A. Methodological notes on studying GxE

We examine the possibility that outcomes associated with the PGS of individual *i* vary as a function of the school *j* that they attend. In general, we assume that the outcome y_{ij} is a function of PGS, x_{ij}, a set of *K* environmental variables e_j^k, and some school-specific quality component u_j. In this framework, outcomes are generated by

$$y_{ij} = \beta_0 + u_j + \beta_1 x_{ij} + f\left(x_{ij}, e_j^k\right) + \epsilon_{ij}. \text{ (Eqn 1)}$$

Note that while there is a main effect (β_1) of the key individual-level variable x_{ij}, the effect of x_{ij} on the outcome may vary as a function of environmental surroundings depending on specifics of f. For example, if

$$f\left(x_{ij}, e_j^k\right) = \theta_1 e_j^1 x_{ij} + \theta_2 e_j^1 e_j^2 x_{ij}$$

then the outcome is a complex function of interactions between the individual-level variable x_{ij} and two environmental variable.

However, Eqn 1 cannot be estimated without measurement of all *K* environmental variables as well as knowledge of $f(x_{ij}, e_j^k)$. One solution is to estimate a tractable alternative that may capture dynamics related to GxE even without specific information about $f(x_{ij}, e_j^k)$. In particular, we consider an indirect approach where we associate with each school a random intercept μ_j and random coefficient δ_j on the PGS term x_{ij}; that is

$$y_{ij} = \alpha_0 + \mu_j + \left(\alpha_1 + \delta_j\right) x_{ij} + \epsilon_{ij} \text{ (Eqn 2)}$$

where

$$\begin{bmatrix} \mu_j \\ \delta_j \end{bmatrix} \sim \text{MVN}\left(\begin{bmatrix} 0 \\ 0 \end{bmatrix}, \begin{bmatrix} \sigma_\mu & \sigma_{\mu\delta} \\ \sigma_{\mu\delta} & \sigma_\delta \end{bmatrix}\right).$$

Here, σ_δ represents variation in the association of the PGS and educational attainment across schools and is our parameter of interest. To illustrate the rationale for this approach, suppose that

f depends on only a single relevant environmental variable. Assuming that this environmental variable has both a main and interactive effect, Eqn 1 becomes

$$y_{ij} = \beta_0 + u_j + \beta_1 x_{ij} + \beta_2 e_j^1 + \beta_3 x_{ij} e_j^1 + \epsilon_{ij}. \text{ (Eqn 3)}$$

Comparing Eqn 2 with Eqn 3, the school-level variation in slopes associated with β_3 in Eqn 3 is captured by σ_δ in Eqn 2 (under certain assumptions regarding the relationship between x_{ij} and e_j^1). Eqn 2 is useful in the absence of knowledge about f whereas Eqn 3 is the typical approach taken in many GxE studies focusing on a single environmental measure; we utilize forms of both in this paper.

B. Methods

B1. Data

The Wisconsin Longitudinal Study (WLS)

The WLS is survey based on a 1/3 sample of all 1957 Wisconsin high school graduates (N=10,317) and a randomly-selected sibling of these graduates (1). The graduate respondents were originally empaneled with an in-person questionnaire at age 18 in 1957, which was followed with data collection at ages 25, 36, 54, 65, and finally 72 in 2012. The paired sibling was randomly selected from a roster of all siblings. The overwhelming European ancestry of WLS respondents matches the ancestry of those on whom the consortia GWAS used for the construction of the PGS, meaning the sample can be used for genetic analyses while minimizing confounding by population stratification. The WLS includes a wide range of administrative and prospectively collected data from early life, adolescence, and early adulthood. For the WLS analysis, we restrict our sample to only European ancestry respondents for whom we have valid genetic data (N~8500). There are over 400 schools represented in our data and these schools generally have forty or fewer genotyped representatives in the WLS, see Figure S1.

The National Longitudinal Study of Adolescent to Adult Health (Add Health)

Add Health is a nationally representative cohort drawn from a probability sample of 80 high schools and 52 middle schools in roughly 80 US communities, and representative of schools in the United States in 1994–95 with respect to region, urban setting, school size, school type, and race or ethnic background (2). Genetic data have recently been collected on a sample of ~10,000 respondents but we focus on a sample of ~4800 unrelated European-descent respondents. Add Health also contains a variety of data on students' academic performance,

employment information, personal characteristics measured in adolescence (cognitive ability, personality characteristics, professional aspirations, physical health and functioning, etc.), and information about the schools attended by the respondents (3). Sample sizes for the 136 schools in our data are shown in Figure S1. The majority of schools have fewer than 100 students, but there are saturation schools in which all students were included in the longitudinal survey (2) that have roughly 400 students represented in the data.

B2. Measures

Here we offer additional detail about the construction of the key measures used in this study. Descriptive statistics for all variables are shown in Table S1. Distributions for the outcomes, along with information on sample size per school, are shown in Figure S1.

Outcomes

- Educational Attainment: Years of completed education measured in Add Health at Wave 4 when respondents were 24-32 years old and in the WLS based on survey responses when respondents were in their 40s.
- Any postsecondary: A binary indicator of whether a subject reported more than 12 years of schooling.
- College Completion: A binary indicator of whether a subject reported 16 or more years of schooling.
- Job Status: Hauser-Warren (4,5) socioeconomic index based on job reported at Wave 4 in Add Health. In WLS, occupational prestige based on job reported in 1992. The construction of these variables is described in detail elsewhere (6).

Predictors

- Polygenic Score: We computed polygenic scores for participants in the Add Health and WLS based on all SNPs analyzed in the most recent Social Science Genetic Association Consortium (SSGAC) GWAS of educational attainment (7). No statistical significance threshold was applied to select SNPs for inclusion in polygenic score analysis. polygenic scores were computed by the SSGAC using the LD Pred software (8). Add Health and WLS were included in the SSGAC GWAS of educational attainment. For each of these datasets, polygenic were computed using summary statistics from GWAS meta-analyses from which the target dataset for polygenic scoring was excluded. Within each dataset,

we regressed SSGAC-computed polygenic scores on the first ten principal components estimated from the genome-wide SNP data (9) and calculated residual values. Finally, we standardized these residual values to have M=0, SD=1 within each dataset to form the final versions of the polygenic scores used for analysis.

- SES: In Add Health, we construct a composite based on the highest reported parental education, parental income, parental job status, and the number of social welfare benefits received. In WLS, a composite based on father's schooling, mother's schooling, father's occupation and parental income. The construction of these variables is described in detail elsewhere (6).
- Adolescent cognition: In Add Health, we use the Peabody picture (10,11) vocabulary test as administered at Wave 1 when respondents were still in secondary school. It has been shown to be associated with a full-scale WAIS score, albeit in a younger sample (12). In WLS, the Henmon-Nelson Test of Mental Ability was administered to WLS participants during high school. It is a 30-minute test consisting of 90 items including vocabulary, sentence completion, disarranged sentences, classification, logical selection, series completion, directions, analogies, anagrams, proverb interpretation, and arithmetic problems (13–15). It highly correlates (r>0.8) with IQ tests more commonly administered today, especially the WAIS (16,17).

Candidate Environments

- School Status: We compute the mean percentage of mothers with at least a high school diploma in the school (i.e., 12 years of schooling). In Add Health, note that this is constructed via student-self report in the full sample of students who participated in the school-based component of the survey (N>90,000). In WLS, this constructed using mother's education of students in our sample.
- School Stratification: We compute the Gini coefficient in the reported levels of educational attainment from which the above measure of parental high school completion is based.

C. Power Analysis

Historically, GxE studies have been underpowered (18). For this reason, we conduct a simulation study to probe our power to detect school-level moderation of the genetic effect. However, it is difficult to conduct an accurate comparison of the statistical power of our direct

and indirect models. This is because the two models test fundamentally different hypotheses. Our indirect model asks: do we observe variation in the association between educational attainment PGS and educational attainment between schools? Our direct model, on the other hand, asks: do we observe variation in the association between educational attainment PGS and educational attainment *as a function of* a specific candidate school environment?

Using the empirically observed school assignment, educational attainment PGS, and occupational attainment from each dataset, we simulate an outcome for a single environmental moderator based on Eqn 3 and β_3 sampled from the uniform distribution on [0,0.1] (note that the main effects used in each simulation are shown in Figure 2). We then estimate both Eqns 2 and 3. We also consider estimates derived in the context of an imperfectly measured proxy for the true environment (where we manipulate the amount of measurement error, based on underlying reliabilities of α=0.4 or α=0.7). We consider 10,000 choices for β_3 with each dataset.

We note here one additional complication of interpreting the results of the power study shown in Figure 2. As mentioned above, because our two models test different hypotheses, comparing the relative statistical power of each model is difficult. When there is only a single environmental variable with a moderating influence, the two hypotheses become identical and Figure 2 provide an accurate comparison of the models. Nonetheless, there may in fact be a multitude of school level-environments that contribute in varying degrees to the moderation of the association of the educational attainment PGS and educational attainment. When multiple environmental moderators are uncorrelated or only weakly correlated, their effects work simultaneously, “stacking” on top of one another and increasing the total between-school variation in PGS slopes. This increase in the available between-school variation for our indirect analyses to detect, in turn, increases its statistical power. At the same time, however, the between-school variation explained by any given candidate environment is relatively unchanged, leaving the statistical power of our direct analyses largely unaffected. Thus, if there are multiple true sources of environmental moderation, the analyses we conducted may underestimate the power of our indirect model.

D. Robustness test of direct analysis in WLS

With respect to the WLS finding, this is perhaps a mechanical GxE in the sense that there is limited variation in educational attainment in low status schools (see Figure 4 of main text). As

a test of this, we estimate Equation 2 of main text separately for the WLS graduates and their siblings. These two groups of respondents had to meet different criteria for inclusion in the WLS; while graduates had to graduate from high school in a certain year to be empaneled, siblings simply had to be a sibling of a WLS graduate, resulting in a less-constrained educational attainment distribution among siblings. To the extent that there is evidence for moderation of educational attainment PGS effect by school status, it is only exists amongst the graduates (Supplement, Table S3), suggesting the results are potentially driven by the truncated distribution of educational attainments.

References

1. Herd P, Carr D, Roan C. Cohort profile: Wisconsin longitudinal study (WLS). Int J Epidemiol. 2014;43(1):34–41.

2. Harris KM. The add health study: Design and accomplishments. Chap Hill Carol Popul Cent Univ N C Chap Hill. 2013;

3. Harris KM, Halpern CT, Hussey J, Whitsel EA, Killeya-Jones L, Tabor J, et al. Social, behavioral, and genetic linkages from adolescence into adulthood. Am J Public Health. 2013;103(S1):S25–S32.

4. Hauser RM, Warren JR. 4. Socioeconomic indexes for occupations: A review, update, and critique. Sociol Methodol. 1997;27(1):177–298.

5. Frederick C, Hauser RM. A crosswalk for using pre-2000 occupational status and prestige codes with post-2000 occupation codes. Cent Demogr Ecol Univ Wis-Madison PMID. 2010;25506974.

6. Belsky D, Domingue B, Wedow R, Arseneault L, Boardman J, Caspi A, et al. Genetic analysis of social-class mobility in five longitudinal studies. Proc Natl Acad Sci. 2018;

7. Lee J, et al. Gene discovery and polygenic prediction from a 1.1-million-person GWAS of educational attainment. Nat Genet. In press;

8. Vilhjálmsson BJ, Yang J, Finucane HK, Gusev A, Lindström S, Ripke S, et al. Modeling linkage disequilibrium increases accuracy of polygenic risk scores. Am J Hum Genet. 2015;97(4):576–592.

9. Price AL, Patterson NJ, Plenge RM, Weinblatt ME, Shadick NA, Reich D. Principal components analysis corrects for stratification in genome-wide association studies. Nat Genet. 2006;38(8):904–909.

10. Dunn LM, Dunn LM. Manual for the peabody picture vocabulary test-revised. Circ Pines MN Am Guid Serv. 1981;

11. Dunn LM, Dunn L, Dunn D. Peabody Picture Vocabulary Test. Circle Pines, MN: American Guidance Service. IncPPVT-III. 1997;

12. Hodapp AF, Gerken KC. Correlations between scores for Peabody Picture Vocabulary Test—III and the Wechsler Intelligence Scale for Children—III. Psychol Rep. 1999;84(3_suppl):1139–1142.

13. Henmon V. Henmon-Nelson Tests of Mental Ability, High School Examination-Grades 7 to 12-Forms A, B, and C. Teacher’s Manual. Boston: Houghton-Mifflin Company; 1946.

14. Henmon VAC. The Henmon-Nelson tests of mental ability. Manual for administration. Chic Houghton-Mifflin. 1954;171:297–318.

15. Henmon VAC, Holt FO. A Report on the Administration of Scholastic Aptitude Tests to 34,000 High School Seniors in Wisconsin in 1929 and 1930: Prepared for the Committee on Cooperation, Wisconsin Secondary Schools and Colleges. Bureau of Guidance and Records of the University of Wisconsin; 1931.

16. Jokela M, Batty GD, Deary IJ, Silventoinen K, Kivimäki M. Sibling analysis of adolescent intelligence and chronic diseases in older adulthood. Ann Epidemiol. 2011;21(7):489–496.

17. Watson CG, Klett WG, Kucala T, Nixon C, Schaefer A, Gasser B. Prediction of the Wais scores from the 1973 Henmon-Nelson revision. J Clin Psychol. 1981;37(4):840–842.

18. Duncan LE, Keller MC. A critical review of the first 10 years of candidate gene-by-environment interaction research in psychiatry. Am J Psychiatry. 2011 Oct;168(10):1041–9.

Table S1. Descriptive Statistics.

	PGS	SES	COG	EDU	Any Postsecondary	College Completion	OCC	Male	Birth-year	School status	School stratification
Add Health						4915 individuals, 136 schools					
mean	-0.001	0.285	105.129	14.227	0.763	0.328	95.949	0.468	78.985	0.472	0.231
sd	1.006	1.178	11.757	2.168	0.426	0.469	37.289	0.499	1.743	0.146	0.034
min	-3.513	-4.402	50.000	8.000	0.000	0.000	21.380	0.000	74.000	0.163	0.077
max	3.472	3.515	138.000	20.000	1.000	1.000	179.510	1.000	83.000	0.937	0.373
n	4915	4716	4703	4915	4915	4915	4831	4915	4914	4725	4725
Correlation with Edu	0.363	0.461	0.377	1.000	0.743	0.803	0.568	-0.123	0.010	0.304	-0.224
Correlation with PGS	1.000	0.290	0.288	0.363	0.260	0.332	0.246	0.022	0.014	0.215	-0.165
ICC	0.065	0.268	0.099	0.160	0.091	0.133	0.077	0.010	0.504		
WLS						8494 individuals, 433 schools, 6432 families					
mean	0.000	16.392	0.118	13.772	0.463	0.293	50.850	0.481	1939.524	0.514	0.138
sd	1.000	11.096	0.979	2.394	0.499	0.455	22.835	0.500	4.288	0.135	0.023
min	-3.610	1.000	-2.614	6.000	0.000	0.000	2.000	0.000	1918.000	0.000	0.043
max	3.259	97.000	2.872	20.000	1.000	1.000	96.000	1.000	1964.000	1.000	0.262
n	8494	8494	7999	8356	8356	8356	7910	8494	8467	8027	8027
Correlation with Edu	0.260	0.397	0.452	1.000	0.829	0.890	0.509	0.142	0.076	0.168	-0.049
Correlation with PGS	1.000	0.128	0.267	0.260	0.224	0.246	0.155	-0.002	-0.010	0.032	0.000
ICC	0.021	0.173	0.070	0.069	0.066	0.056	0.048	0.038	0.007		

Table S2A. Coefficient estimates from Indirect approach (Eqn 1, main text) for school mean centered analyses, Add Health. (AP, Any Postsecondary; CG, College Graduate)

	EDU	EDU	EDU	AP	AP	AP	CG	CG	CG	OCC	OCC	OCC
(Intercept)	0.035	0.066	0.245	-0.431	-0.483	-0.327	-0.126	-0.145	-0.036	0.377	0.543	0.544
(Intercept).se	0.564	0.579	0.578	0.255	0.260	0.259	0.271	0.282	0.282	0.607	0.622	0.623
PGS	0.275			0.082			0.117			0.182		
PGS.se	0.014			0.006			0.007			0.014		
male	-0.246	-0.240	-0.271	-0.082	-0.077	-0.092	-0.087	-0.085	-0.094	-0.253	-0.243	-0.261
male.se	0.025	0.025	0.025	0.011	0.011	0.011	0.012	0.012	0.012	0.027	0.027	0.027
birthyear	0.001	0.001	-0.001	0.006	0.007	0.005	0.002	0.002	0.001	-0.003	-0.005	-0.005
birthyear.se	0.007	0.007	0.007	0.003	0.003	0.003	0.003	0.004	0.004	0.008	0.008	0.008
SES		0.293			0.091			0.122			0.209	
SES.se		0.018			0.007			0.008			0.016	
COG			0.270			0.090			0.100			0.194
COG.se			0.017			0.008			0.008			0.014
SD slopes	0.057	0.131	0.113	0.018	0.033	0.053	0.026	0.054	0.047	0.042	0.077	0.021
SD residual	0.866	0.850	0.859	0.392	0.386	0.385	0.417	0.415	0.420	0.926	0.917	0.927
N	4914	4715	4703	4914	4715	4703	4914	4715	4703	4830	4640	4625
N schools	136	136	136	136	136	136	136	136	136	136	136	136

Table S2B. Coefficient estimates from Indirect approach (Eqn 1, main text) for school mean centered analyses, WLS. (AP, Any Postsecondary; CG, College Graduate)

	EDU	EDU	EDU	AP	AP	AP	CG	CG	CG	OCC	OCC	OCC
(Intercept)	-28.662	-18.668	0.059	-14.348	-10.057	-4.005	-8.526	-4.432	0.972	-11.015	-4.134	7.122
(Intercept).se	4.505	4.448	4.949	2.290	2.261	2.582	2.103	2.085	2.371	4.871	4.839	5.482
PGS	0.221			0.096			0.100			0.130		
PGS.se	0.011			0.006			0.005			0.012		
male	0.241	0.251	0.255	0.089	0.094	0.097	0.087	0.090	0.090	0.087	0.091	0.107
male.se	0.020	0.019	0.019	0.010	0.010	0.010	0.009	0.009	0.009	0.021	0.021	0.021
birthyear	0.015	0.010	0.000	0.007	0.005	0.002	0.004	0.002	-0.001	0.006	0.002	-0.004
birthyear.se	0.002	0.002	0.003	0.001	0.001	0.001	0.001	0.001	0.001	0.003	0.002	0.003
SES		0.262			0.129			0.114			0.178	
SES.se		0.014			0.007			0.006			0.013	
COG			0.358			0.167			0.156			0.304
COG.se			0.012			0.006			0.005			0.011
SD slopes	0.034	0.097	0.080	0.020	0.042	0.037	0.000	0.034	0.025	0.034	0.050	0.013
SD residual	0.782	0.796	0.752	0.400	0.408	0.397	0.372	0.378	0.365	0.889	0.893	0.870
N	8273	8273	7849	8273	8273	7849	8273	8273	7849	7834	7834	7424
N schools	432	432	432	432	432	432	432	432	432	429	429	429
SD family	0.453	0.372	0.359	0.223	0.181	0.174	0.194	0.161	0.160	0.311	0.268	0.237

Table S3. Standardized coefficients of GxE for direct approach based on candidate environments separately for WLS Grads and Sibs (β_3 in Eqn 2, main text).

			Grads			Sibs		
Outcome	Individual	Environment	Estimate	SE	PV	Estimate	SE	PV
Edu	PGS	Status	0.052	0.013	0.000	0.019	0.018	0.303
Edu	PGS	Stratification	-0.009	0.013	0.501	-0.011	0.018	0.550
Any Postsecondary	PGS	Status	0.043	0.014	0.002	0.025	0.018	0.168
Any Postsecondary	PGS	Stratification	0.001	0.014	0.965	-0.033	0.018	0.073
College Completion	PGS	Status	0.047	0.014	0.001	0.027	0.018	0.139
College Completion	PGS	Stratification	-0.006	0.014	0.656	-0.011	0.018	0.566
Occupation	PGS	Status	0.013	0.015	0.373	0.026	0.020	0.190
Occupation	PGS	Stratification	0.001	0.014	0.943	-0.016	0.020	0.424
Edu	SES	Status	0.012	0.013	0.352	0.024	0.015	0.120
Edu	SES	Stratification	0.001	0.013	0.956	-0.002	0.018	0.931
Any Postsecondary	SES	Status	0.003	0.013	0.832	0.010	0.016	0.531
Any Postsecondary	SES	Stratification	-0.002	0.013	0.903	0.018	0.018	0.328
College Completion	SES	Status	0.023	0.013	0.078	0.010	0.015	0.533
College Completion	SES	Stratification	-0.008	0.014	0.547	-0.002	0.018	0.902
Occupation	SES	Status	-0.027	0.013	0.049	0.003	0.017	0.847
Occupation	SES	Stratification	0.012	0.014	0.400	0.007	0.019	0.714
Edu	Cognition	Status	0.045	0.012	0.000	0.019	0.016	0.244
Edu	Cognition	Stratification	-0.004	0.013	0.739	0.007	0.016	0.688
Any Postsecondary	Cognition	Status	0.020	0.013	0.111	0.015	0.017	0.393
Any Postsecondary	Cognition	Stratification	0.007	0.013	0.589	0.009	0.017	0.582
College Completion	Cognition	Status	0.045	0.013	0.000	0.008	0.017	0.648
College Completion	Cognition	Stratification	-0.008	0.013	0.534	0.018	0.017	0.300
Occupation	Cognition	Status	-0.009	0.014	0.503	-0.001	0.019	0.960
Occupation	Cognition	Stratification	0.014	0.014	0.316	0.016	0.019	0.401

Figure S1. Number of respondents per school and density plots/histograms of non-binary outcomes.

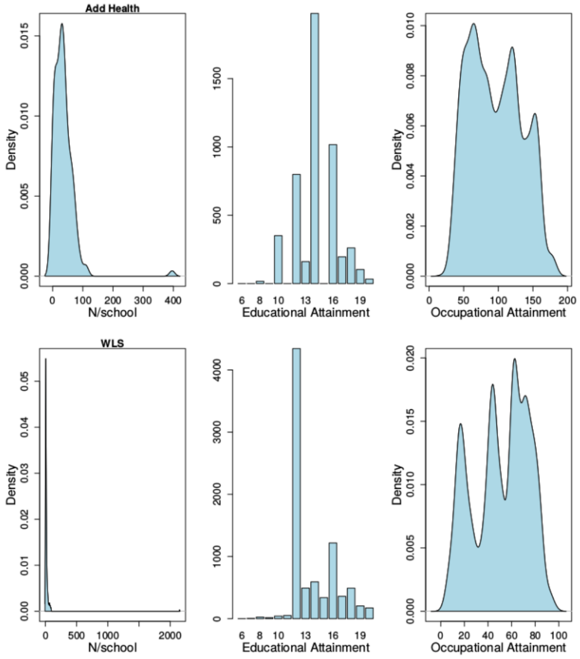

Supplement: Supplemental Material [file NIHMS997312-supplement-Supplemental_Material.pdf]
